# Annual, seasonal, cultural and vacation patterns in sleep, sedentary behaviour and physical activity: a systematic review and meta-analysis

**DOI:** 10.1186/s12889-021-11298-3

**Published:** 2021-07-13

**Authors:** Ty Ferguson, Rachel Curtis, Francois Fraysse, Rajini Lagiseti, Celine Northcott, Rosa Virgara, Amanda Watson, Carol A. Maher

**Affiliations:** Alliance for Research in Exercise, Nutrition and Activity (ARENA), University of South Australia, City East Campus, GPO Box 2471, Adelaide, SA 5001 Australia

**Keywords:** Sleep, Sedentary behaviour, Physical activity, Systematic review, Temporal patterns, Seasonal, Vacation, Cultural patterns, Adults, Time-use

## Abstract

**Background:**

Time spent in daily activities (sleep, sedentary behaviour and physical activity) has important consequences for health and wellbeing. The amount of time spent varies from day to day, yet little is known about the temporal nature of daily activity patterns in adults. The aim of this review is to identify the annual rhythms of daily activity behaviours in healthy adults and explore what temporal factors appear to influence these rhythms.

**Methods:**

Six online databases were searched for cohort studies exploring within-year temporal patterns (e.g. season effects, vacation, cultural festivals) in sleep, sedentary behaviour or physical activity in healthy 18 to 65-year-old adults. Screening, data extraction, and risk of bias scoring were performed in duplicate. Extracted data was presented as mean daily minutes of each activity type, with transformations performed as needed. Where possible, meta-analyses were performed using random effect models to calculate standardised mean differences (SMD).

**Results:**

Of the 7009 articles identified, 17 studies were included. Studies were published between 2003 and 2019, representing 14 countries and 1951 participants, addressing variation in daily activities across season (*n* = 11), Ramadan (*n* = 4), vacation (*n* = 1) and daylight savings time transitions (*n* = 1). Meta-analyses suggested evidence of seasonal variation in activity patterns, with sleep highest in autumn (+ 12 min); sedentary behaviour highest in winter (+ 19 min); light physical activity highest in summer (+ 19 min); and moderate-to-vigorous physical activity highest in summer (+ 2 min) when compared to the yearly mean. These trends were significant for light physical activity in winter (SMD = − 0.03, 95% CI − 0.58 to − 0.01, *P* = 0.04). Sleep appeared 64 min less during, compared to outside Ramadan (non-significant). Narrative analyses for the impact of vacation and daylight savings suggested that light physical activity is higher during vacation and that sleep increases after the spring daylight savings transition, and decreases after the autumn transition.

**Conclusions:**

Research into temporal patterns in activity behaviours is scarce. Existing evidence suggests that seasonal changes and periodic changes to usual routine, such as observing religious events, may influence activity behaviours across the year. Further research measuring 24-h time use and exploring a wider variety of temporal factors is needed.

## Background

Insufficient sleep, excessive sedentary behaviour and insufficient physical activity are all common drivers of weight gain and obesity in adults [1, 2]. These suboptimal daily movement behaviours also increase the risk of many non-communicable diseases (i.e. coronary heart disease, high blood pressure, type 2 diabetes), all-cause mortality, certain cancers, anxiety, and depression [3–9]. Globally, it is estimated 27.5% of people are insufficiently active [10], and a third (32%) report high levels of sedentary behaviour (> 7 h a day) [11], resulting in a global economic burden in 2013 of INT $67.5 billion in health care expenditure and lost productivity [12]. A 2017 multi-country study found the percentage of people reporting insufficient sleep (< 7 h per night) [13] ranged from 26% (Canada) to 56% (Japan) [14].

Substantial resources are put into preventive health each year, including communicable disease control and health promotion (fostering healthy lifestyles, i.e. physical activity) [15]. Current health promotion campaigns typically intervene based on geography, demography (e.g. age or ethnicity) or socioeconomics (e.g. disadvantaged groups) [16–18], yet the timing of health promotion campaign delivery may be overlooked. Temporal factors such as season, cultural and festive periods, and vacation status may influence 24-h movement behaviours. Better understanding temporal patterns in adult movement behaviours may identify new health promotion campaign intervention targets.

Research exploring the temporal factors affecting movement behaviours is scarce. One previous systematic review (*n* = 37 studies) suggested that physical activity varied with season, being lower in winter and higher in summer [19]. However, this study was limited to physical activity only, and involved only narrative synthesis. No other reviews appear to explore temporal factors in movement behaviours. Consistent with contemporary approaches to viewing physical activity along with sedentary behaviour and sleep as forming the 24 h day, this review will consider the impact of temporal factors on all movement behaviours, and use a meta-analytic synthesis, to address the research question: What are the annual rhythms of daily movement behaviours (sleep, sedentary behaviour, light physical activity, and moderate-to-vigorous physical activity) in healthy adults, and what temporal factors appear to influence these?

## Methods

### Protocol and registration

This review follows PRISMA guidelines [20] and was registered on PROSPERO (ID CRD42020184012) [21].

### Eligibility criteria

Studies were included if: they were cohort studies reporting objectively or subjectively measured minutes of sleep, sedentary behaviour, and/or physical activity (light and moderate-to-vigorous); participants were healthy adults aged 18 to 65 years (reflecting the typical age range of adults in health behaviour guidelines); the study explored a temporal pattern (e.g. seasonal effects, pre/post/during vacation or cultural festivals); and the study included at least two assessment time points within a 12-month period using a within-subject design (the most robust design for addressing our research question). Intervention and cross-sectional study designs were excluded along with studies exploring non-general population samples and studies conducted in controlled settings. Studies comparing weekdays to weekends were excluded if only one time point was measured, however longitudinal studies using this comparison were included and weekly means calculated. Studies with populations that spread outside the eligible age range were included if data were able to be extracted (or obtained from authors) for only those participants aged 18–65-years.

### Information sources and search strategy

**Fig. 1:**
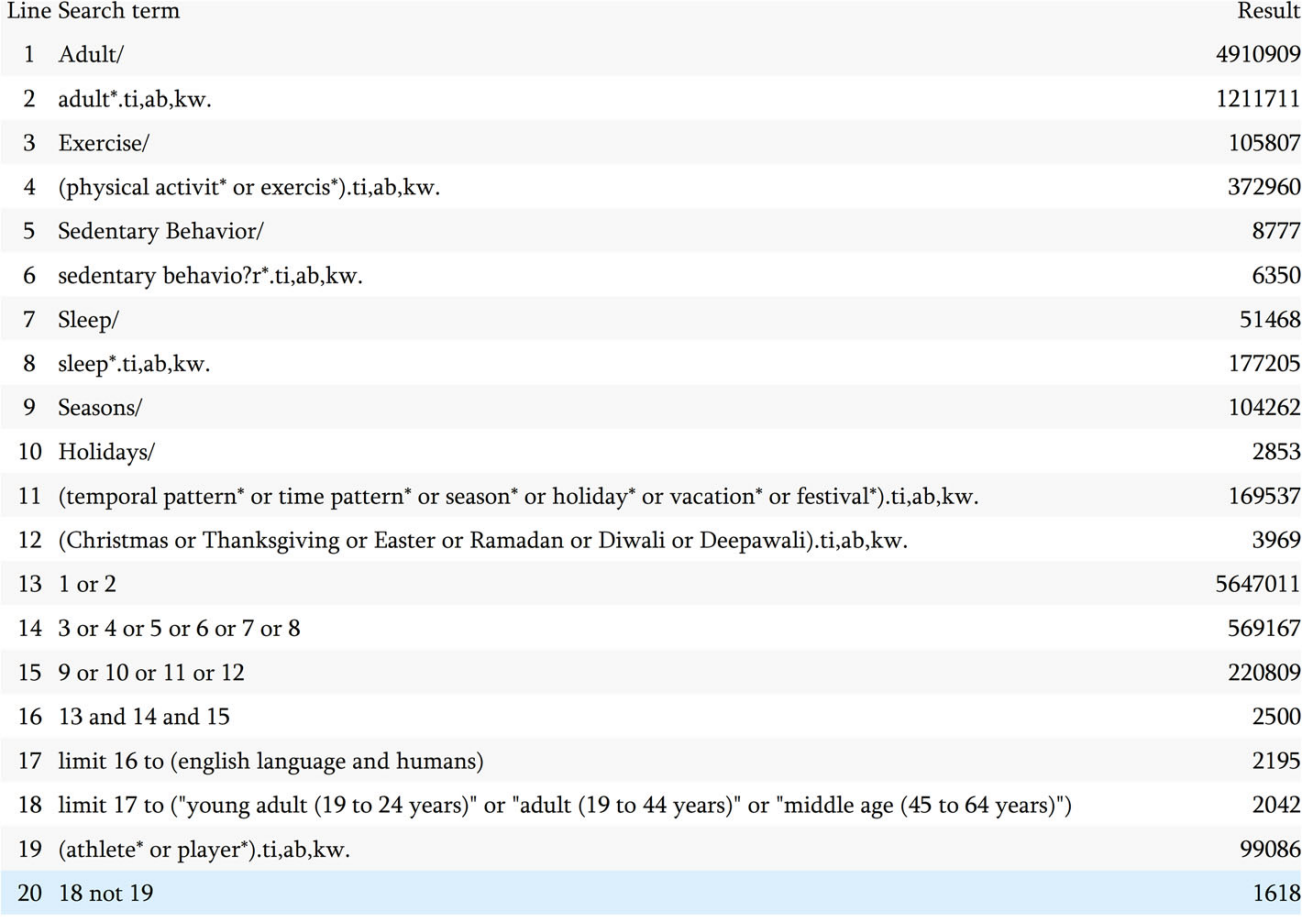
Medline database search terms

The search strategy was developed in consultation with an academic librarian. Six databases were searched in 2020; Embase, Medline, Ovid Emcare, Scopus, Sport Discus, and Web of Science. Temporal patterns included terms relating to seasons, holidays, vacations, and festivals. Specific terms relating to religious events were included for the three most commonly observed religions: Christianity, Islam and Hinduism [22]. Where possible, searches were limited to English language, humans, and adults 18–65 years of age. Athlete* or player* were excluded terms and no limitation was placed on date range. Figure 1 shows the search strategy used for Medline.

### Study selection

All studies were imported into EndNote (EndNote × 9, Clarivate, Philadelphia, USA) for duplicate removal. Remaining studies were exported to Covidence (Covidence systematic review software, Veritas Health Innovation, Melbourne, Australia) for screening. Two independent reviewers completed title and abstract and full-text screening, with a third reviewer consulted for disagreements.

Where necessary, authors of potentially eligible studies were emailed to request additional data. Reference lists of included studies were searched to identify additional studies.

### Data collection

Two independent reviewers used a custom data extraction template, including: study design, exposure (e.g. season, vacation, cultural festival), participant characteristics (sample size, setting, age, sex), outcomes measured (sleep, sedentary behaviour and physical activity), tool used (e.g. accelerometry, questionnaire), assessment time points (duration and frequency), and results (means and standard deviations for daily minutes of each outcome).

### Risk of bias in individual studies

Each study was appraised by two independent reviewers using the Joanna Briggs Institute (JBI) critical appraisal checklist for cohort studies [23]. Conflicts were resolved by a third reviewer. Three items (1, 2 and 6) were irrelevant to single-group study designs and were removed. Each item was rated “yes”, “no”, “unclear”, or “not applicable”. Studies were not excluded based on risk of bias results.

### Data analysis

The primary outcome measure was mean daily minutes spent in sleep, sedentary behaviour, light physical activity and moderate-to-vigorous physical activity. Where moderate and vigorous physical activity were presented separately, the values were added. Where required, data was converted to be expressed as mean daily minutes. This involved combining groups where appropriate (e.g. combining males and females where reported separately), converting monthly data to seasonal data, and converting weekly data to daily data. Standard errors were converted to standard deviations. Where more than 10 studies are identified for an outcome, funnel plots will be used to examine publication bias [24].

Meta-analysis was conducted for outcome and exposures reported in multiple studies. For studies presenting seasonal data, an overall yearly mean (minutes per day) for each outcome was calculated to allow for comparisons with seasonal means. For studies exploring Ramadan, during-Ramadan data were compared with pre/post-Ramadan data. Where multiple data points were reported during or pre/post Ramadan, overall means were calculated by combining relevant data for each condition. A random-effects model was used to calculate standardised mean difference (SMD), pooled SMD, and associated confidence intervals (CI), along with forest plots generated using Review Manager (version 5.4, The Cochrane Collaboration). For seasonal data, estimates of the weighted mean difference (WMD) were calculated as the season mean minus the yearly mean expressed as minutes per day. Each calculation was performed by multiplying the overall yearly standard deviation by the SMD for the season of interest. Estimates of the WMD for during Ramadan compared with outside Ramadan was calculated by multiplying the overall standard deviation for outside-Ramadan by the SMD for during-Ramadan and expressed as minutes per day. Heterogeneity was assessed using the i^2^ statistics, calculated using Review Manager. Analyses were performed in 2020.

## Results

### Included studies

**Fig. 2:**
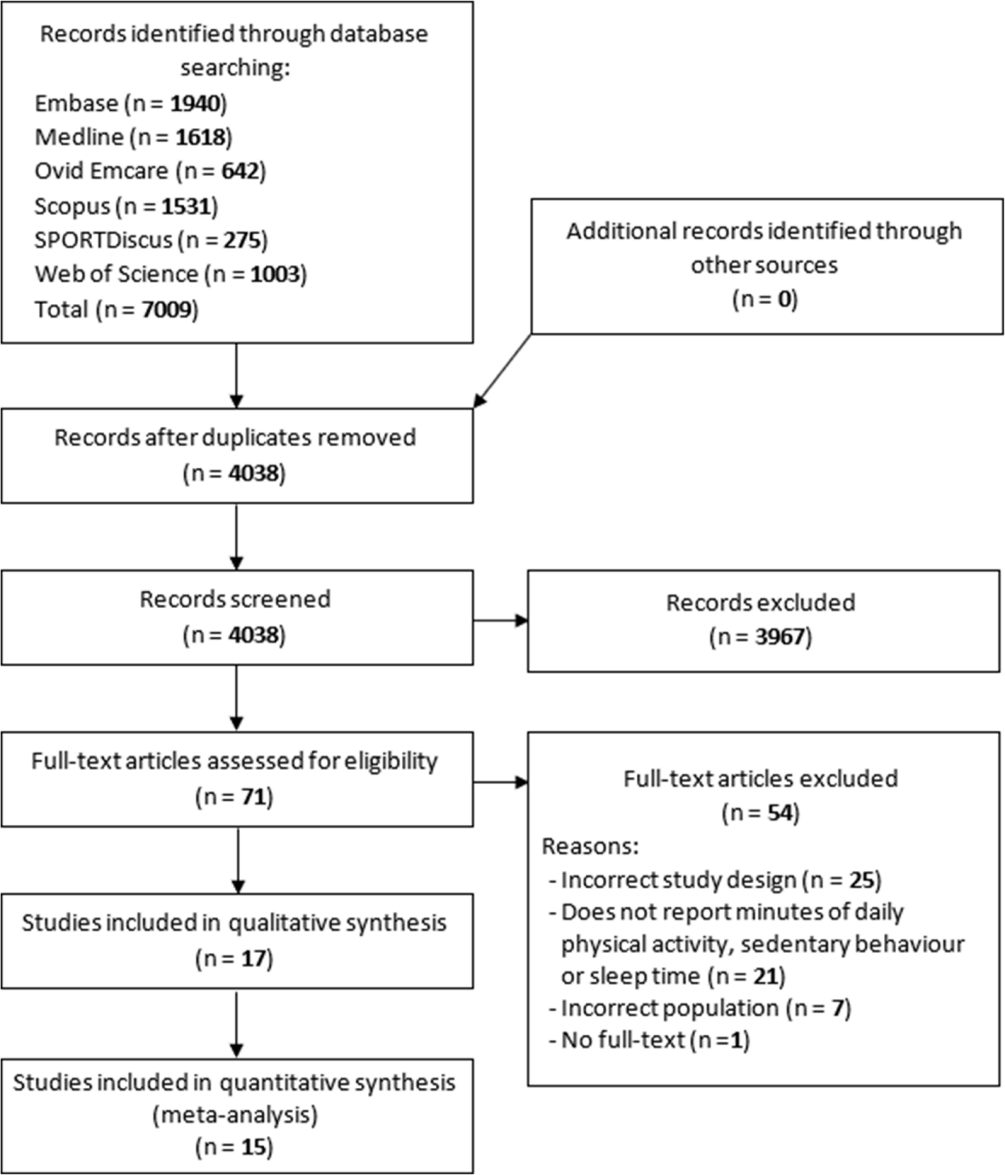
PRISMA flow-chart

The search identified 7009 results, including 4038 different studies, of which 3967 were deemed ineligible based on titles and abstracts. Of the 71 studies reviewed in full text, a further 54 studies were ineligible for several reasons, including 25 had an ineligible study design (e.g. experimental study or cross-sectional), 21 did not report an outcome of interest (minutes of activity), seven had an ineligible population (e.g. children, over 65 yrs., disease populations) and one full-text version of the study was unavailable after extensive searching. Seventeen studies, published between 2003 and 2019, were included (see Fig. 2). Two of the included studies became eligible as a result of contact with authors who provided additional data of interest [25, 26].

### Study characteristics

Table 1 presents a summary of the included studies. Of the 17 included studies, four temporal exposures were present: seasonal patterns (*n* = 11) [26, 32–41], Ramadan (*n* = 4) [28–31] vacation (*n* = 1) [25], and daylight savings time transitions (*n* = 1) [27]. Studies were conducted across four continents: Europe, Africa, Asia, and North America. Most recruited participants from a single country, however, three studies included two or more countries [26, 38, 39]. Multiple studies were conducted in the USA (*n* = 4) [25, 32, 33, 40], Saudi Arabia (*n* = 3) [28–30], Sweden (*n* = 2) [37, 38] and England (*n* = 2) [34, 36], with single studies from Ghana [39], Norway [39], Denmark [38], Germany [35], Czech Republic [26], Slovakia [26], Poland [26], Japan [41], Italy [27], and Libya [31].

**Table 1:** Summary of included studies (*n* = 17)

Study	Temporal pattern	Population	Age- mean (SD), range, % female	Outcome	Tool	Assessment- length, total, frequency
Tonetti 2013 [27]	Daylight savings time	University students, Italy (*n* = 14)	26.9 (3.3), 21–30, 0%	Sleep	Accelerometer: Actiwatch AW-64	7 days × 2, pre/post DST transition
BaHammam 2003 [28]	Ramadan	Fasting Muslim medical students, Saudi Arabia (*n* = 56)	22.6 (1.3), range NR, 45%	Sleep	Questionnaire^a^	5 weekdays ×4, pre/during Ramadan
BaHammam 2005 [29]	Ramadan	Healthy adults, Saudi Arabia (SF *n* = 41 & NSF *n* = 30)	SF: 32.6 (1.5), range NR, 39%. NSF: 29.3 (1.6), range NR, 44%	Sleep	Questionnaire^a^	5 weekdays × 3, pre/during Ramadan
BaHammam 2013 [30]	Ramadan	Male hospital staff, Saudi Arabia (*n* = 8)	36.3 (4.5), range NR, 0%	Sleep	Accelerometer: SenseWear Pro Armband	5 weekdays ×3, pre/during Ramadan
Waterhouse 2009 [31]	Ramadan	Healthy adults, Libya (*n* = 20)	Mean NR, range NR, 0%	Sleep	Questionnaire^a^	1-night × 4, pre/during/post Ramadan
Buchowski 2009 [32]	Season (Wi, Su)	Healthy females, USA (*n* = 57)	36.5 (9.2), 20–54, 100%	SB, LPA, MVPA	Accelerometer: Tritrac-R3D	7 days × 3, consecutive seasons
Pechova 2019 [26]	Season (Sp, Au)	Healthy adults, Czech Republic, Slovakia & Poland (*n* = 50)	61.2 (2.4), 55–65, % NR	SB, LPA, MPA, VPA,	Accelerometer: ActiGraph GT1M	8 days ×2, 6-month follow-up
Lloyd 2013 [33]	Season (Wi, Sp, Su, Au)	Mexican American females, USA (*n* = 36)	33.9 (9.5), 20–63, 100%	MPA, VPA	Questionnaire: National Health and Nutrition Examination Survey	Prior 3 months ×5, quarterly
O’Connell 2014 [34]	Season (Wi, Sp, Su, Au)	Healthy adults, England (*n* = 46)	F: 42.7 (14.8), range NR. M: 39.5 (14.4), range NR, 72%	SB, LPA, MVPA, sleep	Sleep diary^a^, accelerometer: ActiGraph GT1M	7 days × 4, consecutive seasons
Lehnkering 2007 [35]	Season (Sp, Au)	Medical students, Germany (*n* = 34)	Mean NR, 19–31, 56%	Sleep	Accelerometer: Actiwatch, Sleep diary^a^	15 days × 2, 6-month follow-up
Shochat 2019 [36]	Season (Sp, Au)	University students, England (*n* = 19)	18.9 (0.8), range NR, % NR	Sleep	Sleep diary: Karolinska Sleep Diary	21 days × 2, 6-month follow-up
Adamsson 2018 [37]	Season (Wi, Sp, Su, Au)	Daytime office workers, Sweden (*n* = 30)	F: 42.6 (10.0), 24–61, M: 45.2 (14.7), 21–64, 67%	Sleep	Sleep diary^a^	3 weekdays × 12, monthly
Garde 2014 [38]	Season (Wi, Sp, Su, Au)	Hospital staff, Sweden (*n* = 22) & research staff, Denmark (*n* = 16)	Sweden: 48.8 (9.2), 33–62, 73%. Denmark: 42.1 (9.8), 25–62, 63%	Sleep	Sleep diary: Karolinska Sleep Diary, accelerometer: Actiwatch AW4	1 workday night ×12, monthly
Friborg 2012 [39]	Season (Wi, Su)	University students, Ghana (*n* = 200) & Norway (*n* = 200)	Ghana- F: 25.4 (7.5), 19–49, M: 25.3 (6.1), 19–51, 48%. Norway- F: 22.7 (4.8), 19–50, M: 22.7 (5.5), 18–54, 73%	Sleep	Sleep diary^a^	7 days × 2, 6-month follow-up
Ludy 2018 [40]	Season (Wi, Su)	University students, USA (*n* = 60)	18.1 (0.3), range NR, 82%	Sleep	Questionnaire: Youth Risk Behaviour Survey	7 days × 2, 4-month follow-up
Suzuki 2019 [41]	Season (Wi, Su, Sp, Au)	Community residents, Japan (*n* = 960)	YA: mean NR, 19–31, % NR. MA: mean NR, 40–61, % NR	Sleep	Questionnaire^a^	14 nights × 4, consecutive seasons
Cooper 2016 [25]	Vacation	Healthy adults, USA (*n* = 122)	32.2 (13), range NR, 65%	SB, LPA, MPA, VPA	Questionnaire: International Physical Activity Questionnaire	7 days × 3, pre/during/post-vacation

*Au* autumn, *F* female, *LPA* light physical activity, *M* male, *MA* middle-aged, *MPA* moderate physical activity, *MVPA* moderate-to-vigorous physical activity, *NR* not reported, *NSF* Non-Saudi fasting group, *SB* sedentary behaviour, *SF* Saudi fasting group, *Sp* spring, *Su* summer, *VPA* vigorous physical activity, *Wi* winter, *YA* young adult

^a^tool purpose designed for study

### Participants

The studies involved a total of 1951 participants, with sample sizes ranging from 8 [30] to 960 [41], 14 had sample sizes less than *n* = 100. Mean participant age ranged from 18.1 years (SD = 0.3) [40] to 61.2 years (SD = 2.4) [26]. Most studies (*n* = 12) included mixed sex cohorts (range = 39 to 82% female), two had only female participants [32, 33], and three had only males [27, 30, 31].

### Primary outcomes

Sleep was the most common activity examined (*n* = 13) [27–31, 34–41], with sedentary behaviour and light physical activity both measured in four studies [25, 26, 32, 34], and moderate physical activity and vigorous physical activity measured in five studies, either separately or combined as moderate-to-vigorous physical activity [25, 26, 32–34]. Ten studies used subjectively measured activity (i.e. survey, questionnaire, sleep diary) [25, 28, 29, 31, 33, 36, 37, 39–41], four studies used objective measures (accelerometry) [26, 27, 30, 32], and three studies used a combination (accelerometer and sleep diary) [34, 35, 38]. One study used a 24-h accelerometry protocol, allowing changes in sleep, sedentary behaviour, light physical activity and moderate-to-vigorous physical activity to be examined simultaneously [34]. Analyses were not performed to detect potential publication bias due insufficient studies for each outcome and subgroup analyses were not performed due to the limited data available.

### Critical appraisal

Results from the JBI critical appraisal are presented in Table 2. Three studies received ‘yes’ responses for all items [26, 32, 34]. All studies measured the exposure in a valid and reliable way. Almost half of the studies did not identify confounding factors [25, 28–30, 33, 35, 37, 39], nine studies did not state strategies that were used to deal with confounding factors [25, 28–30, 33, 35, 37, 39, 40], whilst it was unclear for one study if strategies were used [31]. It was unclear whether Suzuki et al. [41] and Waterhouse et al. [31] measured outcomes in a valid and reliable way. More than half of the studies had incomplete follow-up [25, 28, 33, 36–41] whilst completeness of follow-up was unclear for one study [31]. Where follow-up was incomplete or unclear, most reported strategies used to address this, however it was unclear for the studies by Garde et al. [38] and Waterhouse et al. [31]. All studies were deemed to have used appropriate statistical analyses.

**Table 2:** Joanna Briggs Institute critical appraisal checklist summary table

	Adamsson 2018 [37]	BaHammam 2003 [28]	BaHammam 2005 [29]	BaHammam 2013 [30]	Buchowski 2009 [32]	Cooper 2016 [25]	Friborg 2012 [39]	Garde 2014 [38]	Lehnkering 2007 [35]	Lloyd 2013 [33]	Ludy 2018 [40]	O’Connell 2014 [34]	Pechova 2019 [26]	Shochat 2019 [36]	Suzuki 2019 [41]	Tonetti 2013 [27]	Waterhouse 2009 [31]
Question	Responses																
3. Was the exposure measured in a valid and reliable way?	✓	✓	✓	✓	✓	✓	✓	✓	✓	✓	✓	✓	✓	✓	✓	✓	✓
4. Were confounding factors identified?	✗	✗	✗	✗	✓	✗	✗	✓	✗	✗	✓	✓	✓	✓	✓	✓	✓
5. Were strategies to deal with confounding factors stated?	✗	✗	✗	✗	✓	✗	✗	✓	✗	✗	✗	✓	✓	✓	✓	✓	U
7. Were the outcomes measured in a valid and reliable way?	✓	✓	✓	✓	✓	✓	✓	✓	✓	✓	✓	✓	✓	✓	U	✓	U
8. Was the follow-up time reported and sufficient to be long enough for outcomes to occur?	✓	✓	✓	✓	✓	✓	✓	✓	✓	✓	✓	✓	✓	✓	✓	✓	✗
9. Was follow up complete, and if not, were the reasons to loss to follow up described and explored?	✗	✗	✓	✓	✓	✗	✗	✗	✓	✗	✗	✓	✓	✗	✗	✓	U
10. Were strategies to address incomplete follow up utilized?	✓	✓	✓	✓	✓	✓	✓	U	✓	✓	✓	✓	✓	✓	✓	✓	U
11. Was appropriate statistical analysis used?	✓	✓	✓	✓	✓	✓	✓	✓	✓	✓	✓	✓	✓	✓	✓	✓	✓

Note – questions 1,2 and 6 omitted as not relevant to included study design

✗ = no, ✓ = yes, U = unclear

### Temporal factors

#### Season

Season was the most examined temporal factor, with 11 studies [26, 32–41].

##### Sleep (*n* = 8 studies)

**Fig. 3:**
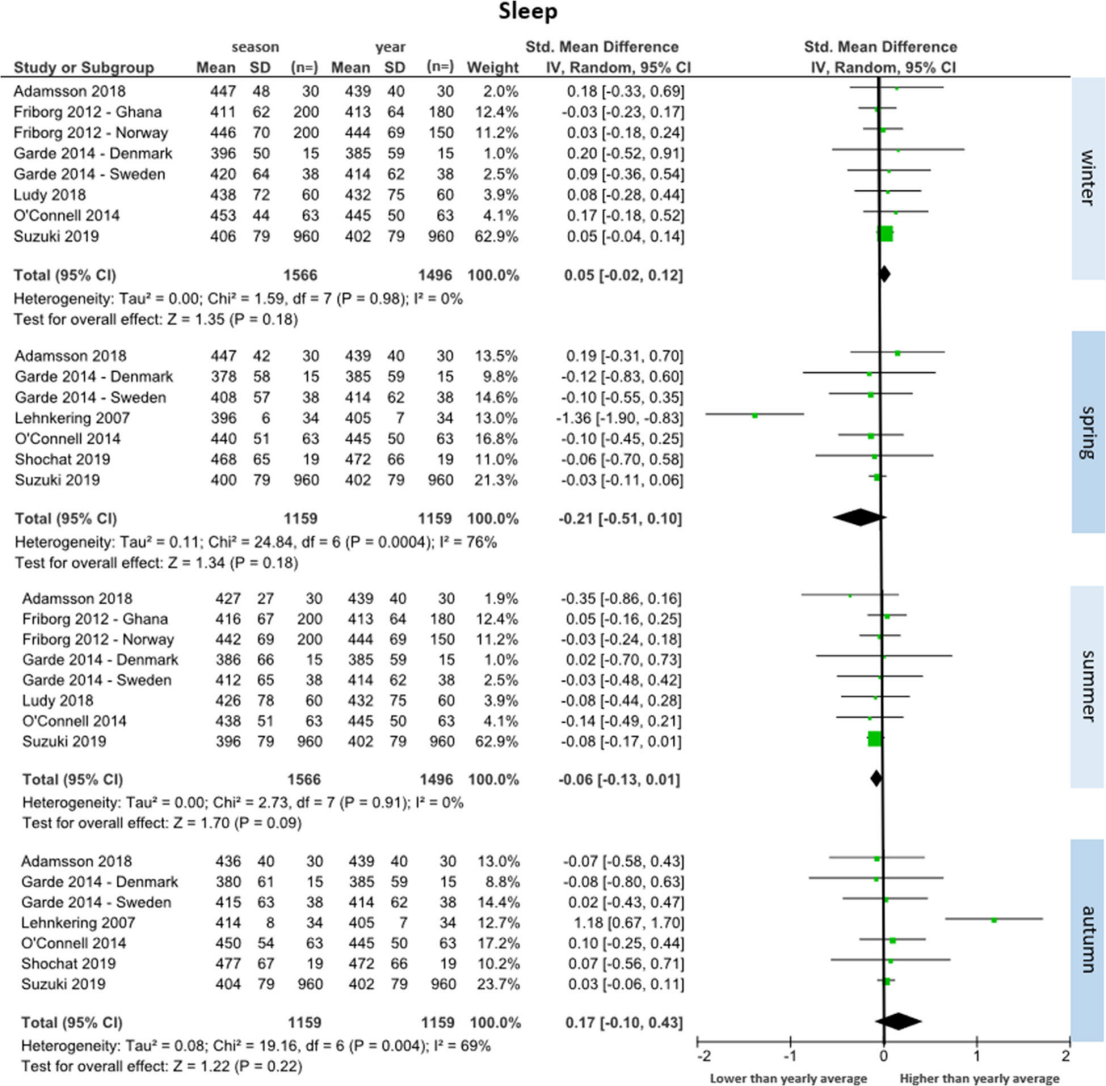
Forest plot of daily minutes of sleep comparing seasonal means with yearly mean

Eight studies reported sleep duration data across seasons (total participants *n* = 1587) [34–41]. Fig. 3 depicts forest plots for sleep in each season compared to the yearly average. Eight studies were included for winter and summer comparisons, and seven for spring and autumn comparisons. No season had significant differences compared to the yearly average. There was a trend towards higher daily minutes of sleep during winter (SMD = 0.05 [95% CI: − 0.02 to 0.12], WMD = 4 min/day) and autumn (SMD = 0.17 [95% CI: − 0.10 to 0.43], WMD = 12 min/day), and less during spring (SMD = − 0.21 [95% CI: − 0.51 to 0.10], WMD = − 15 min/day) and summer (SMD = − 0.06 [95% CI: − 0.13 to 0.01], WMD = − 4 min/day), when compared to the yearly mean. The largest change between seasons was an increase of 27 min/day from spring to autumn. Minimal heterogeneity between studies was observed for winter and summer (I^2^ = 0%) and substantial heterogeneity was observed for spring and autumn (I^2^ = 76 and 69% respectively).

##### Sedentary behaviour (*n* = 3 studies)

**Fig. 4:**
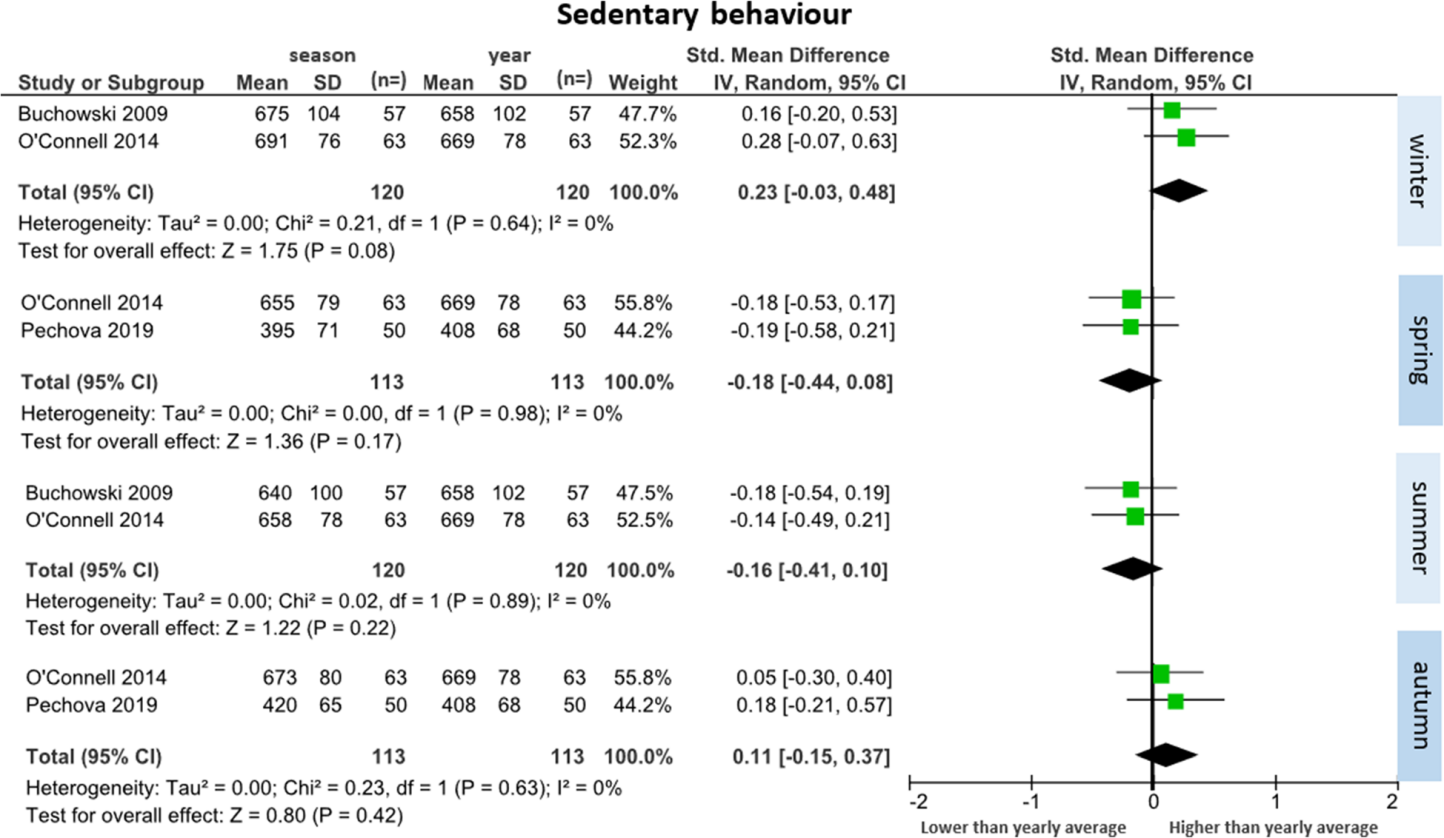
Forest plot of daily minutes of sedentary behaviour comparing seasonal means with yearly mean

Three studies reported sedentary behaviour data across seasons (total participants *n* = 153) [26, 32, 34]. Two studies were included in each comparison (Fig. 4). There were no significant differences in sedentary time in any season compared to the yearly mean. There was a trend towards higher daily minutes of sedentary behaviour during winter (SMD = 0.23 [95% CI: − 0.03 to 0.48], WMD = 19 min/day) and autumn (SMD = 0.11 [95% CI: − 0.15 to 0.37], WMD = 9 min/day), and less during spring (SMD = − 0.18 [95% CI: − 0.44 to 0.08], WMD = − 15 min/day) and summer (SMD = − 0.16 [95% CI: − 0.41 to 0.10], WMD = − 14 min/day), when compared to the yearly mean. The largest change between seasons was an increase of 36 min/day from spring to winter. Minimal heterogeneity between studies was observed across all seasons (I^2^ = 0%).

##### Light physical activity (*n* = 3 studies)

**Fig. 5:**
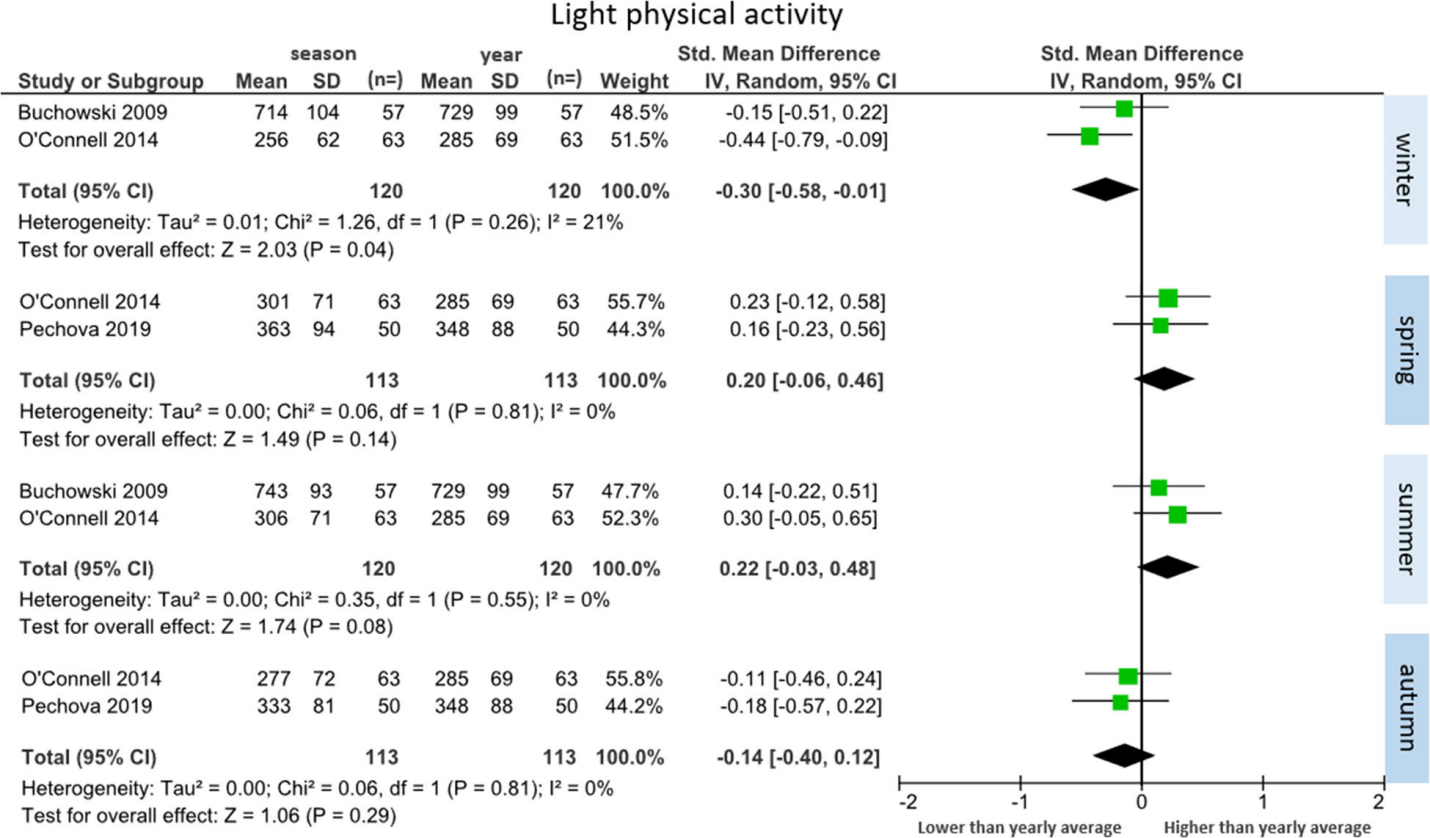
Forest plot of daily minutes of light physical activity comparing seasonal means with yearly mean

Three studies reported light physical activity data across seasons (total participants *n* = 153) [26, 32, 34]. Two studies were included in each comparison (Fig. 5). Significantly less light activity occurred during winter compared to the yearly mean (SMD = − 0.30 [95% CI: − 0.58 to − 0.01], WMD = − 26 min/day). No significant differences were found with the remaining seasons. There was a trend towards more light physical activity during spring (SMD = 0.20 [95% CI: − 0.06 to 0.46], WMD = 17 min/day) and summer (SMD = 0.22 [95% CI: − 0.03 to 0.48], WMD = 19 min/day), when compared to the yearly average, and less light physical activity in autumn (SMD = − 0.14 [95% CI: − 0.40 to 0.12], WMD = − 12 min/day). The largest change between seasons was an increase of 45 min/day from winter to summer. Minimal heterogeneity between studies was observed across all seasons (winter: I^2^ = 21%; spring, summer and autumn: I^2^ = 0%).

##### Moderate-to-vigorous physical activity (*n* = 4 studies)

**Fig. 6:**
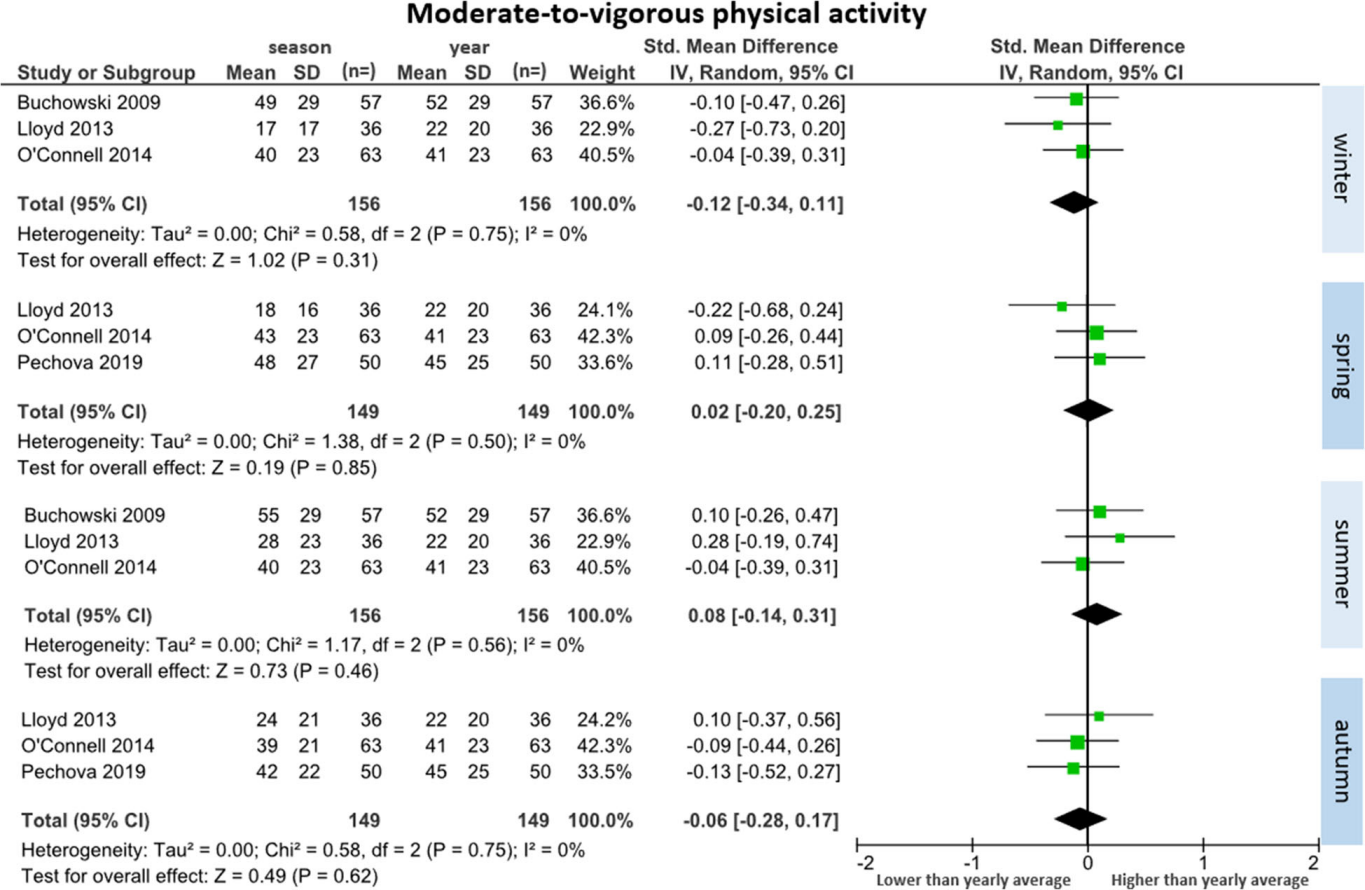
Forest plot of daily minutes of moderate-to-vigorous physical activity comparing seasonal means with yearly mean

Four studies reported moderate-to-vigorous physical activity data across seasons (total participants *n* = 189) [26, 32–34]. Three studies were included in each comparison (Fig. 6). There were no significant differences in any season compared to the yearly average. There was a trend toward less moderate-to-vigorous physical activity during winter (SMD = − 0.12 [95% CI: − 0.34 to 0.11], WMD = − 3 min/day) and autumn (SMD = − 0.06 [95% CI: − 0.28 to 0.17], WMD − 1 min per day). There was trend toward more moderate-to-vigorous physical activity during summer (SMD = 0.08 [95% CI: − 0.14 to 0.31], WMD = 2 min/day) and minimal difference from the yearly average for spring (SMD = 0.02[95% CI: − 0.20 to 0.25], WMD = 0.5 min/day). The largest change between seasons was an increase of 5 min/day from winter to summer. Minimal heterogeneity between studies was observed across all seasons (I^2^ = 0%).

#### Ramadan (*n* = 4 studies)

**Fig. 7:**
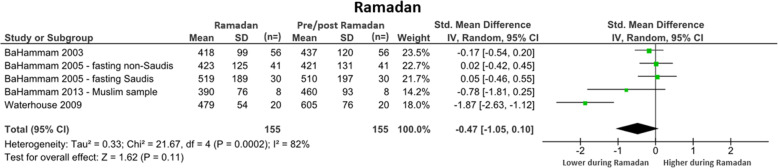
Forest plot of daily minutes of sleep comparing during Ramadan with pre/post-Ramadan

Four studies explored daily activity patterns across Ramadan. All measured sleep duration only. For control, studies used either the week before Ramadan [28–30], or the mean of a night before and after Ramadan [31]. The overall SMD was non-significant when comparing sleep duration during Ramadan to pre/post Ramadan (SMD = − 0.47 [95% CI: − 1.05 to 0.10]). There was a trend towards less sleep during Ramadan than on the control nights (WMD = 64 min/day). Substantial heterogeneity was observed between studies (I^2^ = 82%). See Fig. 7 for further detail.

#### Vacation (*n* = 1 study)

One study [25] explored the effect of vacationing away from home on sedentary behaviour, light physical activity and moderate-to-vigorous physical activity. Participants engaged in significantly more daily minutes of light physical activity on vacation than before (104 min [SD = 88 min] vs.71 min [SD = 74 min], *p* < 0.001)) and after vacation (104 min [SD = 88 min] vs. 68 min [SD = 73 min], p < 0.001)). This study reported a statistically non-significant trend for participants to have engaged in less daily minutes of sedentary behaviour (215 min [SD = 99 min]) compared with before (252 min [SD = 132 min]) and after vacation (251 min [SD = 131 min]), and a statistically non-significant trend to have engaged in less daily minutes of moderate-to-vigorous physical activity during vacation (29 min (SD = 35 min) compared to before (50 min [SD = 81 min]) and after vacation (41 min [SD = 46 min]).

#### Daylight savings time (*n* = 1 study)

One study [27] explored the impact of the change in daylight savings time on sleep, measuring sleep time at both the autumn transition out of daylight savings time and the spring transition into daylight savings time. In the week after the autumn transition out of daylight savings time, participants slept significantly less (25 min per day less, *p* < 0.05) than during daylight savings (365 min vs 340 min). In the week after the spring transition into daylight savings time, participants slept significantly more (28 min per day more, p < 0.05) than before daylight savings (340 min vs 368 min).

## Discussion

### Principal findings

This systematic review has brought together current evidence regarding annual rhythms in adults’ daily movement behaviours and examined key temporal factors which may influence these rhythms. Results reveal only a handful of studies identified per temporal factor, mostly involving small sample sizes. Regardless, several trends emerged. Sleep appeared to be higher during autumn and winter, outside of Ramadan and after the transition into daylight savings time. Sedentary behaviour also appeared higher during autumn and winter, and during non-vacation periods. Light physical activity was higher during spring and summer and whilst on vacation, and moderate-to-vigorous physical activity appeared to be higher during spring and summer, and during non-vacation periods.

Seasonal effects on movement behaviours (particularly sleep) was the most commonly explored temporal factor. The meta-analyses were generally non-significant (except for light physical activity), however methodological factors may have contributed to this. Many studies used self-reported tools, which are known to have modest validity [42] and may in turn have limited the ability to accurately detect change over time. Secondly, study populations were small (especially for the sedentary behaviour, light physical activity and MVPA meta-analyses). However, the direction for change in movement behaviours across seasons were generally consistent across studies, and varied in the expected directions, suggesting that the non-significant trends may be real.

Results suggested sleep duration was approximately half-an-hour less in summer compared with autumn. This may influence health outcomes, particularly if sleep totals fall outside the optimal range for positive health outcomes of 7-8 h [43]. For example a one-hour decrease in sleep below 7 h is associated with a 6% increase in all-cause mortality, an 11% increase in cardiovascular disease risk [43], and a 9% increase in obesity risk [44]. Results suggested that sedentary behaviour is around 30 min higher in winter, relative to spring. It is questionable whether this would impact health. However, it was coupled with lower light physical activity (45 min less, relative to summer) and less moderate-to-vigorous physical activity (5 min less, relative to summer), which is likely to be of sufficient magnitude to have health consequences. For example, a 1 min per day increase in moderate-to-vigorous physical activity has been associated with a 2% decrease in all-cause mortality [45]. It has been suggested that as little as 15 min extra light physical activity, or 5 min extra moderate-to-vigorous physical activity, has a positive effect on all-cause mortality and cardiovascular disease [46].

It is important to consider that season interacts with climate and daylight hours differently in different geographic regions. Almost all seasonal studies included in this review took place in temperate climate locales in the mid-latitudes (between 23^o^ and 66^o^) of the northern hemisphere. Temperate climates are characterised by mild-to-warm summers and cool-to-cold winters with moderate yearly rainfall [47]. Mid-latitudes have distinct differences in daylight hours between seasons of approximately 4-8 h between winter and summer. Only a single study sample originated in a tropical climate and latitude (Ghana) [39]. Given the small number of studies in this review, it is not possible to disentangle the effects of season, climate and daylight hours. Temperate climates dominate the evidence presented here, and further research in tropical and subpolar regions is warranted.

Whilst this review sought studies examining various cultural, religious and festive periods’, all eligible studies of this type related to Ramadan only, and its impacts on sleep. Ramadan is an example of a periodic change to typical lifestyle patterns. Specifically, Ramadan is a spiritual disciplinary practice, where people observing Ramadan do not eat or drink during daylight hours, changing usual mealtimes, along with usual bedtimes and wake times, and therefore changes in movement behaviours could be expected. Additionally, during this month, people are reportedly less active during daylight hours [48] however none of the included studies examined physical activity during Ramadan. Across studies, participants observing Ramadan generally went to bed and woke later, with the delay in bedtime more pronounced than wake-time. Whilst there was a trend towards less sleep during Ramadan, the weighted mean sleep duration of 7 h and 25 min falls within recommended levels of 7 or more hours per night [13]. It should be noted that Ramadan is the ninth month of the Islamic calendar, which is 10–12 days shorter than the Gregorian calendar, therefore over several years, Ramadan will occur across all seasons. It is possible the trends observed in sleep time across seasons may interact with Ramadan differently depending on the season, with shorter winter days allowing for an extended window to consume meals, impacting less on usual sleep times. Of the studies exploring Ramadan, three were conducted in Saudi Arabia, and one in Libya. Both countries are located at a similar latitude (approximately 16-33^o^), experiencing similar daylight hours across the year. Across the four included studies, Ramadan occurred between late Summer and mid-winter. While changes to sleep routine during Ramadan may be unaffected by seasonal changes at latitudes with small fluctuations in daylight hours across the year, future research in different locations is warranted.

The single study exploring daylight savings transitions reported a significant change in sleep duration of approximately half-an-hour after each transition, with less in autumn and more in spring. It is worth noting duration at all time points fell below recommended levels of sleep per night of 7 or more hours [13]. The immediate one-hour shift in clock time requires an adjustment period for our internal biological clocks to reset to the new timing of daylight hours, accounting for the immediate disruptions to sleep duration [49]. It is likely the observed changes in sleep duration return to pre-transition levels in following weeks, however further research for longer periods is needed to examine this. Further studies are needed before conclusions can be made about the impact of daylight savings transitions.

The final periodic temporal factor explored was vacation periods where again, only a single study was found. This study suggested participants on vacation got less moderate-to-vigorous physical activity and more light physical activity and sedentary time. Further research is needed to allow for the impact of vacations on movement behaviours to be understood. Additionally, future studies should explore movement behaviour intentions for vacation periods as it may be a risk factor for poor movement behaviour during vacation. The location or time of year when participants went on vacation was not reported, and thus it is important to consider that season may be a confounding factor.

### Implications

This review identified trends towards negative changes in daily movement behaviours during several periods. Results suggest physical activity interventions may be needed during winter and autumn months where activity levels decline. Efforts could focus on alternate exercise options for these periods, such as indoor or home-based exercise routines, along with the provision of indoor or sheltered environments within the community to combat the environmental barriers during these colder, wetter and darker periods. Interventions promoting regular movement during winter and autumn could also target sedentary behaviour. Periodic temporal factors such as observing religious practices have potential to provide spiritual, physical and/or mental health benefits which would suggest these periods may not be the optimal time for movement behaviour intervention. Further research is needed to understand whether there are significant and lasting effects on usual movement behaviours resulting from periodic temporal factors. Opportunities may exist to promote pre-emptive behaviour changes, to counteract the effects of these periodic temporal factors if those effects are found to be impactful on health. For example, increased physical activity could be encouraged in the lead up to vacation or moving bedtime earlier before the autumn daylight savings transition.

### Strengths and limitations

This is the first systematic review and meta-analysis of its kind. Several strengths to this systematic review include:, the search strategy was rigorous and designed in consultation with an academic librarian, and followed PRISMA guidelines [20]; study screening, data extraction and risk of bias were all performed in duplicate, increasing accuracy and trustworthiness of results.

A key limitation of the review was the relatively small number of studies exploring each of the four included temporal factors, ranging from one study each for daylight savings time and vacation, to 11 studies for season. Most studies had small sample sizes (*n* = 60 or less in 14 studies). The sleep meta-analyses were dominated by a single study with a comparatively large sample (*n* = 960). Only one of the included studies reported the 24-h day, with a large portion of studies measuring sleep only. Limiting to English language studies may have reduced the variety of temporal patterns explored, specifically cultural or religious events in non-English speaking countries. Only a limited number of countries were included, each with their own seasonal and daylight hour patterns, therefore findings may not be representative of countries not explored in this review.

### Future recommendations

This systematic review highlights the limited number of cohort studies exploring annual patterns of activity. Future research with larger sample sizes, in more diverse geographic areas, and using high quality movement behaviour measurement methods, are needed. Very little research has examined changes in daily movement behaviours during festive and cultural periods. Additionally, no studies have explored the interaction between temporal factors. Changes in a particular behaviour must be necessarily compensated by a change in another behaviour, given that there are 24 h in a day. Methodologies which measure multiple movement behaviours simultaneously, ideally using a 24-h measurement approach, are needed.

## Conclusions

This study examined current research exploring annual rhythms in adults’ daily movement behaviours and the key temporal factors which may influence these rhythms. Several trends were observed across each temporal factor including more sleep in autumn, winter and outside Ramadan, more sedentary behaviour in autumn and winter, and more light physical activity and moderate-to-vigorous physical activity in spring and summer. These trends highlight potential at-risk periods of negative movement behaviours (decline in sleep and physical activity, increase in sedentary behaviour) which may inform the timing and aid the design of health promotion and interventions strategies. Only single studies for daylight saving transitions and vacation were included, making it difficult to draw conclusions on the impact of these temporal factors. The current evidence for temporal patterns of movement behaviours and the association with temporal factors is scarce. Future research should aim to explore the 24-h day of movement behaviours using larger samples from a greater diversity of geographic areas, along with exploring a wider variety of temproal factors that may influence these behaviours.

## Data Availability

The datasets used and/or analysed during the current study are available from the corresponding author on reasonable request.

## Abbreviations

PRISMA: Preferred Reporting Items for Systematic Reviews and Meta-Analyses
SMD: Standardised mean difference
WMD: Weighted mean difference
